# Covariance of pairwise differences on a multi-species coalescent tree and implications for *F*_ST_

**DOI:** 10.1098/rstb.2020.0415

**Published:** 2022-06-06

**Authors:** Geno Guerra, Rasmus Nielsen

**Affiliations:** ^1^ Department of Statistics, University of California, Berkeley, CA 94720, USA; ^2^ Department of Integrative Biology, University of California, Berkeley, CA 94720, USA; ^3^ Department of Neurological Surgery, University of California, San Francisco, CA 94158, USA; ^4^ Lundbeck Foundations Centre for GeoGenetics, University of Copenhagen, Kobenhavn, Denmark

**Keywords:** multi-species coalescent, covariance, *F*_ST_, population differentiation, pairwise differences

## Abstract

The multi-species coalescent (MSC) provides a theoretical foundation for modern phylogenetics and comparative population genetics. Its theoretical properties have been heavily studied but there are still aspects of the MSC that are largely unknown, including the covariances in pairwise coalescence times, which are fundamental for understanding the properties of statistics that combine data from multiple species, such as the fixation index (*F*_ST_). The major contribution of this study is the derivation and implementation of exact expressions for the covariances of pairwise coalescence times under phylogenetic models with piecewise constant changes in population size, assuming no gene flow after species divergence. We use these expressions to derive the variance in average pairwise differences within and between populations. We then derive approximations for the expectation and bias of a sequence-based estimator of *F*_ST_, a commonly used genetic measurement of population differentiation, when it is applied to a non-recombining region of the genome. We show that the estimator of *F*_ST_ is generally biased downward. A freely available software package is provided, STCov, to calculate the mean, variances and covariances in coalescence times presented here under user-defined piecewise-constant species trees.

This article is part of the theme issue ‘Celebrating 50 years since Lewontin's apportionment of human diversity’.

## 1. Introduction

The multi-species coalescent (MSC) is a generalization of Kingman’s coalescent [1] that describes the joint coalescence process in multiple species, or populations, as they diverge from each other. The MSC provides a theoretical foundation for phylogenetic analyses as it fully describes and characterizes the process of incomplete lineage sorting [2–5]. It is, therefore, central in the unification of the fields of population genetics and phylogenetics. It is also central for understanding divergence between populations and allows the theoretical prediction of the amount of variance within and between populations. In this sense, it provides a theoretical framework for relating apportionment of genetic variance within and between populations, as proposed by Lewontin [6], to specific models of population divergence.

One of the important utilities of theoretical models, such as the MSC, is to provide predictions regarding observed statistics, eventually leading to the development of estimators of population-level parameters. In this regard, an important use of the MSC has been to understand the properties of pairwise nucleotide differences within and between species, which is one of the most commonly used statistics to analyse population genetic data. Takahata & Nei [7] derived expressions for the variance in average pairwise nucleotide differences and Nei and Li’s ‘net number of differences’ [8], (*d*). They assumed a Kingman’s coalescent model [1] of two diverging populations, and an infinite sites model of mutation [9,10]. These classical results provided insights into when the net number of differences can be used as a reliable estimator for species divergence, and the appropriate sampling schemes to reduce the variance. It is also one of the first uses of the MSC.

Takahata & Nei [7] defined *d*_*X*_ and *d*_*Y*_ to be the mean number of nucleotide differences between two (haploid) individuals sampled from within population *X* or *Y*, respectively. Similarly, *d*_*XY*_ is the average number of nucleotide differences between two individuals randomly sampled from populations *X* and *Y*. The statistics *d*_*X*_, *d*_*Y*_ and *d*_*XY*_ are then calculated based on sample sizes of *n*_*X*_ and *n*_*Y*_ from populations *X* and *Y*, respectively, as follows:

1.1 $$d_{X}=\frac{2}{n_{X}(n_{X}-1)}\sum_{i=1}^{n_{X}-1}\sum_{i^{\prime }=i+1}^{n_{X}}k_{i,i^{\prime }}$$

1.2 $$d_{Y}=\frac{2}{n_{Y}(n_{Y}-1)}\sum_{i=1}^{n_{Y}-1}\sum_{i^{\prime }=i+1}^{n_{Y}}k_{\;j,j^{\prime }}$$

1.3 $$\mathrm{and}\;d_{XY}=\frac{1}{n_{X}n_{Y}}\sum_{i=1}^{n_{X}}\sum_{\;j=1}^{n_{Y}}k_{i,j},$$

where *k*_*i*,*i*′_ is the number of pairwise nucleotide differences between individuals (haplotype genomic sequences) *i* and *i*′. Henceforth, in this study, an ‘individual’ is a non-recombining haploid genomic sequence.

To measure the net number of nucleotide differences between two populations, Nei & Li’s [8] *d* is defined as

1.4 $$d=d_{XY}-\frac{1}{2}(d_{X}+d_{Y}).$$

The relationship between differences within and between populations gives an indication of the degree of population subdivision. *d* specifically measures the excess number of substitutions between populations, which quantifies the extent of divergence. These measures of species divergence form the basis for many evolutionary analyses and are among the most basic and commonly used inferential tools in modern population genetics.

The pairwise differences *d*_*XY*_, *d*_*X*_ and *d*_*Y*_ provide measures of genetic variability within and between species/populations that are applicable to DNA sequencing data and have been fundamental in analyses of such data since the 1980s. However, since their invention, the question quickly arose of how they relate to older measures of genetic divergence and variability originally derived for independent loci such as allozymes, in particular, how are they related to Wright’s *F*_ST_? Furthermore, how should *F*_ST_ appropriately be calculated for DNA sequencing data? These questions were answered by Slatkin [11], who argued that *F*_ST_ is equivalent to a ratio of average coalescence times of different pairs of genes. Assuming an infinite sites model, he then showed that Wright’s *F*_ST_ in the context of DNA sequencing data could be expressed in terms of *d*_*XY*_, *d*_*X*_, and *d*_*Y*_ (see equation (7.2) below).

The statistics *d*_*XY*_, *d*_*X*_ and *d*_*Y*_ have been, and continue to be, a cornerstone of the analysis of DNA sequence data. Understanding their mean, variances and covariances under arbitrary genetic and species tree models is essential for their biological interpretability, and considerable previous work has been devoted to understanding their properties. Tajima [12] and Takahata & Nei [7] studied the variance of average pairwise differences in a panmictic population and in a split model with constant population size. In a series of papers, Wakeley studied the variance in pairwise differences in a general model of population sub-division [13] and the average pairwise differences in a model with migration [14], and later demonstrated the impact of recombination on the numerical stability of such estimates [15]. Tang *et al.* [16] derived an estimator for the time to most recent common ancestor (TMRCA) of a sample of DNA sequences along with quantification of sampling error by leveraging pairwise differences, free of population structure assumptions.

The multi-species coalescent has received renewed attention in the age of genomics because of its applicability in phylogenetic analyses using multiple loci. Efromovich & Kubatko [17] presented a method to calculate the distribution of coalescent times at the root of a species tree with an arbitrary number of populations. In a pair of papers, Wilkinson-Herbots provided unified analytic results for both the distribution of coalescence times and pairwise differences under models of isolation with migration [18,19] under assumptions of constant population size. Heled [20] helped to further marry previously pairwise difference quantification and the multispecies coalescent by deriving closed-form exact results for the ‘average sequence dissimilarity’ between pairs of sequences drawn at random under a simple two-species coalescent process with constant population size. Many methods have also been developed to use pairwise differences under the MSC while leveraging large genomics datasets to infer species tree topologies and divergence times (e.g. [21–23]).

Takahata & Nei’s [7] original results on *d*_*XY*_, *d*_*X*_ and *d*_*Y*_ relied on the assumption of constant and equal population sizes among populations and through time. Using the MSC, we here extend these results to arbitrary piecewise constant population size histories along a phylogeny. To do so, we derive and present general equations for calculating the covariance of pairwise coalescence times, for any two, three or four haploid individuals, arbitrarily chosen within the phylogeny. We also derive expressions for the expected shared branch length between sets of lineages. We provide a software package, STCov, for calculating these theoretical MSC quantities. We then use these results to demonstrate the effects of various demographic, mutational and sampling size changes on the distribution of *d*, and extend the discussion to specifically investigate the statistical properties of Slatkin’s *F*_ST_ estimator [11], and some of its various applications [24–26], as it is the most commonly used measure of *F*_ST_ using sequence data. We investigate the effects of bottlenecks, sampling variance and demographic changes on various *F*_ST_-based measurements, and present the magnitude of downward bias when using *F*_ST_ estimated from a ‘ratio of averages’ approach to Slatkin’s estimator, as is typical in single gene analyses.

## 2. Mean, variance and covariance of average pairwise differences

We first review previous results for the mean, variance and covariance of average pairwise nucleotide differences for individuals sampled from two populations, *X* and *Y*, as functions of the individual pairwise difference terms (*k*_*i*,*i*′_, *k*_*i*,*j*_ · · ·). Suppose *i*, *i*′, *i*″, *i*″′ are individuals from population *X*, and *j*, *j*′, *j*″, *j*″′ are individuals from population *Y*. By definition we have,

2.1 $$E(d_{X})=E(k_{i,i^{\prime }}),$$

and likewise for population *Y*. Suppose *i*, *j* are individuals from *X*, *Y*, respectively, then,

2.2 $$E(d_{XY})=E(k_{i,j}).$$

Following the derivations in Tajima [12], Takahata & Nei [7] and Wakeley [14], under an infinite-site model of mutation, the variance and covariance of *d*_*X*_, *d*_*Y*_, *d*_*XY*_ and *d* can be written as follows:

2.3 $$\begin{array}{rl} \text{Var}(d_{X}) & =\frac{1}{n_{X}(n_{X}-1)}[2E(k_{i,i^{\prime }}^{2})+4(n_{X}-2)E(k_{i,i^{\prime }}k_{i,i^{\prime \prime }})\\ & \;+(n_{X}-2)(n_{X}-3)E(k_{i,i^{\prime }}k_{i^{\prime \prime },i^{\prime \prime \prime }})]-E{\left(k_{i,i^{\prime }}\right)}^{2}, \end{array}$$

2.4 $$\begin{array}{rl} \text{Var}(d_{Y}) & =\frac{1}{n_{Y}(n_{Y}-1)}[2E(k_{\;j,j^{\prime }}^{2})+4(n_{Y}-2)E(k_{\;j,j^{\prime }}k_{\;j,j^{\prime \prime }})\\ & \;+(n_{Y}-2)(n_{Y}-3)E(k_{\;j,j^{\prime }}k_{\;j^{\prime \prime },j^{\prime \prime \prime }})]-E{\left(k_{\;j,j^{\prime }}\right)}^{2}, \end{array}$$

2.5 $$\begin{array}{rl} \mathrm{Var}(d_{XY}) & =\frac{1}{n_{X}n_{Y}}[E(k_{i,j}^{2})+(n_{Y}-1)E(k_{i,j}k_{i^{\prime },j})+(n_{X}-1)E(k_{i,j}k_{i,j^{\prime }})\\ & \;+(n_{X}-1)(n_{Y}-1)E(k_{i,j}k_{i^{\prime },j^{\prime }})]-E{\left(k_{ij}\right)}^{2} \end{array}$$

2.6 $$\begin{array}{rl} \mathrm{and}\;\mathrm{Var}(d) & =\mathrm{Var}(d_{XY})+\frac{1}{4}\left[\mathrm{Var}(d_{X})+\mathrm{Var}(d_{Y})\right]\\ & \;+2\mathrm{Cov}(d_{X},d_{Y})-\mathrm{Cov}(d_{XY},d_{X})]\\ & \;-\mathrm{Cov}(d_{XY},d_{Y}). \end{array}$$

Further, formulae for the covariance of average pairwise difference terms can also be reduced to functions of individual pairwise terms

2.7 $$\mathrm{Cov}(d_{X},d_{Y})=\mathrm{Cov}(k_{i,i^{\prime }},k_{\;j,j^{\prime }}).$$

This simple result is due to the fact that the covariance of sums can be decomposed into the sums of covariances.

As presented in Takahata & Nei (equations 18a–d) [7], covariance equations involving the cross population can be expressed as follows:

2.8 $$\mathrm{Cov}(d_{XY},d_{X})=\frac{2}{n_{X}}E(k_{i,i^{\prime }}k_{i,j})+\frac{n_{X}-2}{n_{X}}E(k_{i,i^{\prime }}k_{i^{\prime \prime },j})-E(k_{i,i^{\prime }})E(k_{\;j,j^{\prime }})$$

and

2.9 $$\mathrm{Cov}(d_{XY},d_{Y})=\frac{2}{n_{Y}}E(k_{\;j,j^{\prime }}k_{i,j})+\frac{n_{Y}-2}{n_{Y}}E(k_{\;j,j^{\prime }}k_{i,j^{\prime \prime }})-E(k_{i,i^{\prime }})E(k_{\;j,j^{\prime }}).$$

These expressions are all functions of the individual pairwise differences, e.g. *k*_*i*,*i*′_. In what proceeds we demonstrate that these expressions can be further generalized as functions of pairwise coalescence times, e.g. *t*_*i*,*i*′_.

## 3. Pairwise mutational differences

In this section, we generalize previous work [7,12] by deriving expressions for the covariance of pairwise differences under arbitrary piecewise-constant demographic settings using the MSC. Throughout this section, we will assume an infinite sites model [9,10], with no recombination. We first review results on the mean and variance from previous work (e.g. [7,12,14]), and then extend results to the covariance.

### (a) Mean and variance

Note, given a coalescence time *t*_*i*,*j*_ between two individuals, *i* and *j*, the expected number of nucleotide differences between the pair is equal to 2*μt*_*i*,*j*_, for i.e.

3.1 $$E(k_{i,j})=2\mu E(t_{i,j}).$$

Under the assumption that the number of mutations conditional on a genealogy is Poisson, the conditional expectation and variance of pairwise differences are equal.

3.2 $$\text{Var}(k_{i,j}|t_{i,j})=E(k_{i,j}|t_{i,j}).$$

By applying the law of total variance, we can decompose the unconditional variance of pairwise differences as

3.3 $$\begin{array}{rl} \sigma_{k_{i,j}}^{2}=\mathrm{Var}(k_{i,j}) & =E\left(\mathrm{Var}(k_{i,j}|t_{i,j})\right)+\mathrm{Var}\left(E(k_{i,j}|t_{i,j})\right)\\ & =E(2\mu t_{i,j})+\mathrm{Var}(2\mu t_{i,j})\\ & =2\mu E(t_{i,j})+4\mu^{2}\mathrm{Var}(t_{i,j}). \end{array}$$

We can obtain the second moment of the distribution of pairwise nucleotide differences, $E(k_{i,j}^{2})$, from the definition of variance,

3.4 $$E(k_{i,j}^{2})=\sigma_{k_{i,j}}^{2}+E{\left(k_{i,j}\right)}^{2}=2\mu E(t_{i,j})+8\mu^{2}E{\left(t_{i,j}\right)}^{2}.$$

### (b) Covariance

Let *i*, *i*′, *j*, *j*′ be four individuals sampled from arbitrary populations. Let *T* be a local coalescent tree relating the four individuals restricted to a non-recombining region. Here, we show that

3.5 $$\text{Cov}(k_{i,i^{\prime }},k_{\;j,j^{\prime }}|T)=\mu t_{i,i^{\prime }\cap j,j^{\prime }}.$$

Consequently, we further derive the unconditional quantity

**Figure 1.**
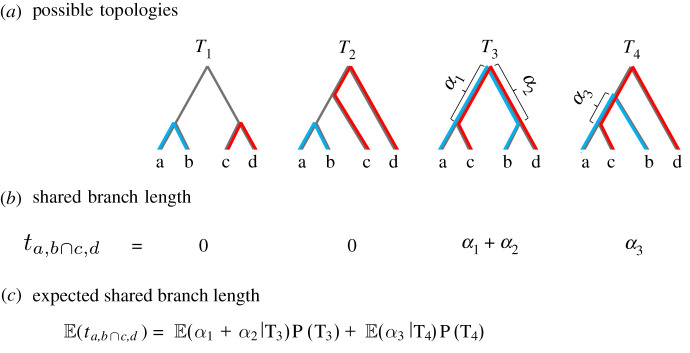
(*a*–*c*) Explanation of expected shared branch length for four unique individuals. Bolded blue lines indicate the branch length between individuals a and b. Bolded red lines indicate branch length between c and d. Overlapping blue and red lines (along with *α* terms) indicate shared branch length. The four tree topologies are representative of the possible gene tree orderings, but it should be noted that these representative trees assume a and b are exchangeable, as well as c and d. The expected shared branch length is a weighted sum of the shared branch lengths across all possible topology orderings. (Online version in colour.)

3.6 $$\mathrm{Cov}(k_{i,i^{\prime }},k_{\;j,j^{\prime }})=\mu E(t_{i,i^{\prime }\cap j,j^{\prime }})+4\mu^{2}\mathrm{Cov}(t_{i,i^{\prime }},t_{\;j,j^{\prime }}),$$

where $t_{i,i^{\prime }\cap j,j^{\prime }}$ denotes the amount of branch length on *T* shared between the branch connecting pair *i*, *i*′ and the branch connecting pair *j*, *j*′. Figure 1 provides an illustrative example of this quantity, and electronic supplementary material, §F, provides a more technical treatment.

To prove these results, we start by revisiting the idea that under the infinite-site model, the mutational process given a branch length is Poisson. Given local tree, *T*, with coalescence times *t*_*i*,*i*′_ and *t*_*j*,*j*′_ from *T*, conditional pairwise differences follow a Poisson distribution, written as

$$k_{i,i^{\prime }}|t_{i,i^{\prime }}∼\text{Poisson}(2\mu t_{i,i^{\prime }})\;\text{ and }\;k_{\;j,j^{\prime }}|t_{\;j,j^{\prime }}∼\text{Poisson}(2\mu t_{\;j,j^{\prime }}),$$

where 2*t*_*i*,*i*′_ is the amount of total branch length locally between the two individuals. A key feature of the Poisson distribution is that the sum of Poisson random variables is also Poisson. To exploit this, let $t_{i,i^{\prime }\cap j,j^{\prime }}$ denote the amount of branch length on *T* shared by pairs *i*, *i*′ and *j*, *j*′ (figure 1). The branch length between *i*, *i*′ not shared with pair *j*, *j*′ is denoted by $t_{i,i^{\prime }\setminus j,j^{\prime }}$, with similar notation for pair *j*, *j*′ by swapping labels. We can decompose the branch lengths into the shared and non-shared segments as

3.7 $$2t_{i,i^{\prime }}=t_{i,i^{\prime }\cap j,j^{\prime }}+t_{i,i^{\prime }\setminus j,j^{\prime }}\;\text{and}\;2t_{\;j,j^{\prime }}=t_{i,i^{\prime }\cap j,j^{\prime }}+t_{\;j,j^{\prime }\setminus i,i^{\prime }}.$$

Notice that $k_{i,i^{\prime }\cap j,j^{\prime }}|T$, $k_{i,i^{\prime }\setminus j,j^{\prime }}|T$ and $k_{\;j,j^{\prime }\setminus i,i^{\prime }}|T$ are therefore independent Poisson random variables. Similarly, $k_{i,i^{\prime }}=k_{i,i^{\prime }\cap j,j^{\prime }}+k_{i,i^{\prime }\setminus j,j^{\prime }}$ and $k_{\;j,j^{\prime }}=k_{i,i^{\prime }\cap j,j^{\prime }}+k_{\;j,j^{\prime }\setminus i,i^{\prime }}$, where $k_{i,i^{\prime }\cap j,j^{\prime }}$, $k_{\;j,j^{\prime }\setminus i,i^{\prime }}$ and $k_{i,i^{\prime }\setminus j,j^{\prime }}$ are independent of each other conditionally on *T*.

We can expand Cov(*k*_*i*,*i*′_, *k*_*j*,*j*′_|*T*), (equation 3.5) as follows:

$$\begin{array}{rl} \mathrm{Cov}(k_{i,i^{\prime }},k_{\;j,j^{\prime }}|T) & =\mathrm{Cov}(k_{i,i^{\prime }\cap j,j^{\prime }}+k_{i,i^{\prime }\setminus j,j^{\prime }},k_{i,i^{\prime }\cap j,j^{\prime }}+k_{\;j,j^{\prime }\setminus i,i^{\prime }}|T)\\ & =\mathrm{Var}(k_{i,,i^{\prime }\cap j,j^{\prime }}|T)+\mathrm{Cov}(k_{i,i^{\prime }\cap j,j^{\prime }},k_{i,i^{\prime }\setminus j,j^{\prime }}|T)\\ & \;+\mathrm{Cov}(k_{i,i^{\prime }\cap j,j^{\prime }},k_{\;j,j^{\prime }\setminus i,i^{\prime }}|T)+\mathrm{Cov}(k_{i,i^{\prime }\setminus j,j^{\prime }},k_{\;j,j^{\prime }\setminus i,i^{\prime }}|T)\\ & =\mathrm{Var}(k_{i,i^{\prime }\cap j,j^{\prime }}|T)\\ & =\mu t_{i,i^{\prime }\cap j,j^{\prime }}. \end{array}$$

The overall result is that the covariance of pairwise differences given the coalescent tree *T* is equal to the mutation rate times the shared branch length.

To get the unconditional quantity, Cov(*k*_*i*,*i*′_, *k*_*j*,*j*′_) (equation 3.6), we apply the law of total covariance:

$$\begin{array}{rl} \mathrm{Cov}(k_{i,i^{\prime }},k_{\;j,j^{\prime }}) & =E\left(\mathrm{Cov}(k_{i,i^{\prime }},k_{\;j,j^{\prime }}|T)\right)+\mathrm{Cov}\left(E(k_{i,i^{\prime }}|T),E(k_{\;j,j^{\prime }}|T)\right)\\ & =E(\mu t_{i,i^{\prime }\cap j,j^{\prime }})+\mathrm{Cov}(2\mu t_{i,i^{\prime }},2\mu t_{\;j,j^{\prime }})\\ & =\mu E(t_{i,i^{\prime }\cap j,j^{\prime }})+4\mu^{2}\mathrm{Cov}(t_{i,i^{\prime }},t_{\;j,j^{\prime }}). \end{array}$$

The case when for only three unique individuals (*k*_*i*,*i*′_, *k*_*i*,*j*_) has the same form, by replacing *j*′ with *i* in the equations above.

Takahata & Nei [7] have previously derived formulas for the covariance under constant population size; see electronic supplementary material, §C, which presents a visualization of their results as a comparison to the generalized results presented here.

## 4. Mean, variance and covariance in pairwise coalescence times

**Figure 2.**
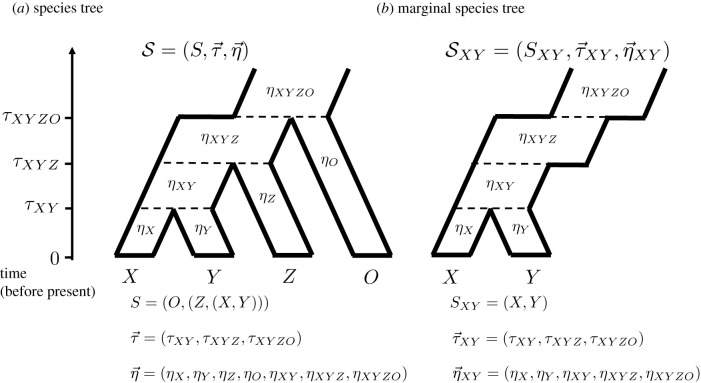
Species tree notation. (*a*) Example of notation used for a four-species tree with topology, divergence times and constant population sizes within each population which can vary between species. (*b*) Example of a marginal species tree, the result of subsetting a larger species tree. As a consequence, the population size histories are no longer constant within each species, but instead are piecewise constant.

We assume species evolution follows a bifurcating species tree $S=(S,\vec{\tau },\vec{\eta })$, with no migration (see figure 2*a*). Each branch, *i*, of S is parameterized by constant diploid population size *η*_*i*_, start time *τ*_*i*_, and end time *τ*_*p*(*i*)_, where *p*(*i*) is the parent branch of *i*. Let *μ* be the mutation rate (constant across the genome/species) per sequence per generation. Time is measured in units of generations in the past. We implicitly assume that all coalescent calculations here are conditioned on a fixed species tree S, although the tree is not always indicated in the notation for the sake of simplicity and compactness.

### (a) Mean and variance in coalescence times

Let *t*_*i*,*j*_ be the coalescence time of two individuals, *i* and *j,* sampled from species *X* and *Y*, respectively, in a non-recombining region of the genome. For species tree S, denote the marginal tree $S_{XY}=(S_{XY},{\vec{\tau }}_{XY},{\vec{\eta }}_{XY})$ of two species (see figure 2*b*). Here, ${\vec{\tau }}_{XY}$ represents the set of divergence times of species ancestral to both *X* and *Y*, indexed by (*τ*_1_, *τ*_2_, …), where *τ*_1_ : = *τ*_*XY*_, the divergence time for species *X* and *Y*. Similarly, ${\vec{\eta }}_{XY}$ represents the corresponding population sizes. Suppose there are *V* ≥ 1 intervals in $S_{XY}$.

Under this marginal tree, we can analytically calculate the first two moments of the distribution of *t*_*i*,*j*_ as

4.1 $$\begin{array}{rl} E(t_{i,j}|S) & =\sum_{k=1}^{V}P_{22}(\tau_{1},\tau_{k})\int_{\tau_{k}}^{\tau_{k+1}}t_{i,j}P(t_{i,j}|S,\tau_{k})\;dt_{i,j}\\ & =\sum_{k=1}^{V}P_{22}(\tau_{1},\tau_{k})\int_{\tau_{k}}^{\tau_{k+1}}\frac{t_{i,j}}{2\eta_{k}}\;e^{-(\left(t_{i,j}-\tau_{k}\right)/2\eta_{k})}\;dt_{i,j}\\ & =\sum_{k=1}^{V}P_{22}(\tau_{1},\tau_{k})\left[-(\tau_{k+1}+2\eta_{k})\;e^{-((\tau_{k+1}-\tau_{k})/2\eta_{k})}+\tau_{k}+2\eta_{k}\right] \end{array}$$

and

4.2 $$\begin{array}{rl} E(t_{i,j}^{2}|S) & =\sum_{k=1}^{V}P_{22}(\tau_{1},\tau_{k})\int_{\tau_{k}}^{\tau_{k+1}}t_{i,j}^{2}P(t_{i,j}|S,\tau_{k})\;dt_{i,j}\\ & =\sum_{k=1}^{V}P_{22}(\tau_{1},\tau_{k})\int_{\tau_{k}}^{\tau_{k+1}}\frac{t_{i,j}^{2}}{2\eta_{k}}\;e^{-(\left(t_{i,j}-\tau_{k}\right)/2\eta_{i})}\;dt_{i,j}\\ & =\sum_{k=1}^{V}P_{22}(\tau_{1},\tau_{k})[-(\tau_{k+1}^{2}+4\tau_{k+1}\eta_{k}+8\eta_{k}^{2})\;e^{-(\left(\tau_{k+1}-\tau_{k}\right)/2\eta_{k})}\\ & \;+\tau_{k}^{2}+4\tau_{k}\eta_{k}+8\eta_{k}^{2}]. \end{array}$$

*P*_22_(*τ*_1_, *τ*_*k*_) represents the probability that lineages *i* and *j* fail to coalesce in the time interval (*τ*_1_, *τ*_*k*_), (two lineages in, two lineages out). Formally, this is the probability that two lineages which exist in the same population at time interval *τ*_1_ have not coalesced by time *τ*_*k*_ (backwards in time)

4.3 $$P_{22}(\tau_{1},\tau_{k})=\prod_{\tau_{1}\leq \tau_{l}<\tau_{k}}\;e^{-(\left(\tau_{l+1}-T_{l}\right)/2\eta_{l})}.$$

Note that the mean $E(t_{i,j}|S)$ and variance $\mathrm{Var}(t_{i,j}|S)=E(t_{i,j}^{2}|S)-E{\left(t_{i,j}|S\right)}^{2}$ of pairwise coalescence times under the standard piecewise constant coalescent process are just simply weighted sums over coalescence intervals.

### (b) Covariance in pairwise coalescence times

The challenge in calculating the covariance terms from a species tree, S, comes from the combinatorial problem of integrating over all of the possible times and orderings of the coalescent events along the multi-species tree. The general formula for covariance in this case is given by

$$\mathrm{Cov}(t_{i,i^{\prime }},t_{\;j,j^{\prime }}|S)=E(t_{i,i^{\prime }}t_{\;j,j^{\prime }}|S)-E(t_{i,i^{\prime }}|S)E(t_{\;j,j^{\prime }}|S),$$

where the last term is simply a product of independent expectations. The first term on the right-hand side of the equation is what we will focus on; in particular, we write

4.4 $$\begin{array}{r} E(t_{i,i^{\prime }}t_{\;j,j^{\prime }}|S)=\int_{D_{\;j,j^{\prime }}}^{\infty }t_{\;j,j^{\prime }}P(t_{\;j,j^{\prime }}|S)\int_{D_{i,i^{\prime }}}^{\infty }t_{i,i^{\prime }}P(t_{i,i^{\prime }}|t_{\;j,j^{\prime }},S)\;dt_{i,i^{\prime }}\;dt_{\;j,j^{\prime }}. \end{array}$$

*D*_*i*,*i*′_ is the species divergence time between individuals *i*, *i*′ from S, where *D*_*i*,*i*′_ = 0 if *i*, *i*′ are of the same species (similarly for *D*_*j*,*j*′_). We assume all coalescence events must be at least as ancient as the species divergence time (e.g. *t*_*j*,*j*′_ ≥ *D*_*j*,*j*′_), i.e. we assume no introgression, migration or admixture, etc.

To evaluate this quantity, $E(t_{i,i^{\prime }}t_{\;j,j^{\prime }}|S)$, we consider six separate conditional cases. For a bifurcating tree of four individuals, there are three unique coalescence events. The six cases correspond to the possible orderings of coalescence events for this local tree of four individuals, given that we structure the joint likelihood as $P(t_{i,i^{\prime }}|t_{\;j,j^{\prime }},S)P(t_{\;j,j^{\prime }}|S)$:

*C*_1_. *t*_*i*,*i*′_ is the first coalescent event.
*C*_2_. *t*_*i*,*i*′_ is the second event, *t*_*j*,*j*′_ is the third.
*C*_3_. *t*_*i*,*i*′_ = *t*_*j*,*j*′_ as the third coalescent event.
*C*_4_. *t*_*j*,*j*′_ is the second event, *t*_*i*,*i*′_ is the third.
*C*_5_. *t*_*j*,*j*′_ is the first event, *t*_*i*,*i*′_ is the second.
*C*_6_. *t*_*j*,*j*′_ is the first event, *t*_*i*,*i*′_ is the third.

**Figure 3.**
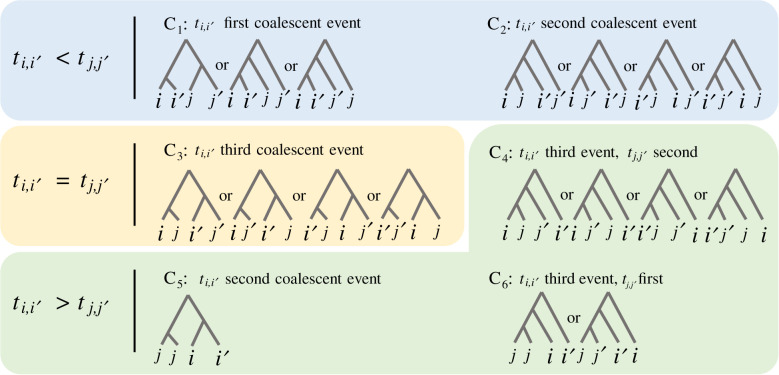
Ordered topologies to consider when calculating *t*_*i*,*i*′_|*t*_*j*,*j*′_. Given four individuals, *i*, *i*′, *j*, *j*′, the six cases presented outline the necessary labelled/ordered local trees essential for the conditional calculation of *P*(*t*_*i*,*i*′_|*t*_*j*,*j*′_). The cases can be grouped into three general scenarios based on the timing of *t*_*i*,*i'*_ in relation to the conditional *t*_*j*,*j*′_. All 18 possible ordered tree topologies are considered. (Online version in colour.)

Here, ‘first event’ implies most recent, and ‘third’ implies most ancient. These events are further illustrated in detail in figure 3. Conditioning on each of these six events, and evaluating each expectation separately, the expression for the joint expectation becomes

4.5 $$E(t_{i,i^{\prime }}t_{\;j,j^{\prime }}|S)=\sum_{k=1}^{6}E(t_{i,i^{\prime }}t_{\;j,j^{\prime }}|S,C_{k})P(C_{k}|S).$$

In the presence of no population isolation (all individuals from the same species), but piecewise constant population size history, the set of recursions and integrals is presented in its entirety in the electronic supplementary material, §G. This calculation is useful in the instance that all four lineages survive to a common population without having coalesced with one another, which occurs with some probability in each case.

Introducing a species tree structure on top of the six cases multiplies the number of cases to consider. There are five general possible species tree configurations that can arise (see electronic supplementary material, figure S13). We have derived exact equations and recursions to evaluate all six cases (*C*_1_, …, *C*_6_) across the five general possible tree configurations, and have implemented them in C++ code (STCov) which is freely available to use (more information in the code availability section). From this implementation, we are able to calculate exact theoretical quantities for these statistics under any piecewise constant scenario.

## 5. Accuracy of coalescent calculations

**Figure 4.**
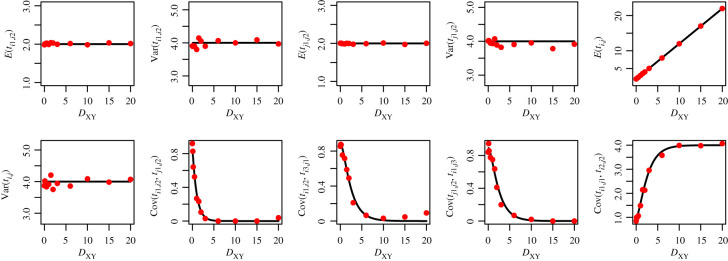
Assessing the accuracy of theoretical pairwise coalescent time calculations against simulated values, for population sizes: *η*_*Y*_ = *η*_*X*_. Theoretical results from STCov are plotted as black curves, with dots representing empirical estimates of the quantity on the *y*-axis using 4500 independently simulated local trees.

**Figure 5.**
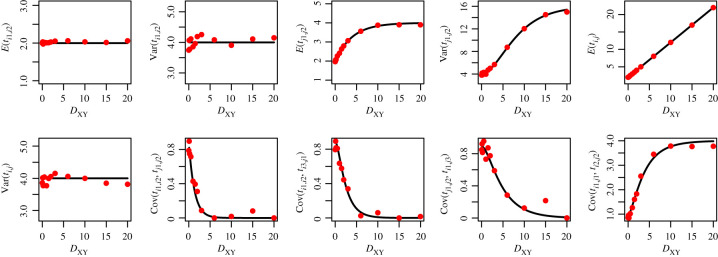
Assessing the accuracy of theoretical pairwise coalescent time calculations against simulated values, for population sizes: *η*_*Y*_ = 2*η*_*X*_. Theoretical results from STCov are plotted as black curves, with dots representing empirical estimates of the quantity on the *y*-axis using 4500 independently simulated local trees. (Online version in colour.)

To demonstrate the accuracy of the coalescent equations above, as implemented in our software STCov, we compare the theoretical results (assuming infinite-sites) against empirical estimates from gene trees under a finite-sites model using ms [27]. We first test two simple demographic scenarios for a tree of two species, *X* and *Y*: *η*_*Y*_ = *η*_*X*_, and *η*_*Y*_ = 2*η*_*X*_ (figures 4 and 5), where *η* represents scaled effective population size. We assume *η*_*XY*_ = *η*_*X*_ in both scenarios. Let lineages *i*_1_, *i*_2_, *i*_3_ originate in population *X*, and lineages *j*_1_, *j*_2_, *j*_3_ originate in *Y*. We generate 1500 independent gene trees from ms for each demographic scenario (with specified population sizes and single divergence time which we vary from 0–20 in units of 2*η*_*X*_ generations), and calculate sample mean, variance and covariance terms. The figures demonstrate that the theoretical calculations from STCov match simulations (dots) well, while variation in the empirical estimates can be attributed to a finite sample size.

## 6. Accuracy of pairwise difference calculations

In this section, we evaluate the accuracy of our results under varying mutation rates, divergence times and population sizes. We compare our results to simulated datasets.

**Figure 6.**
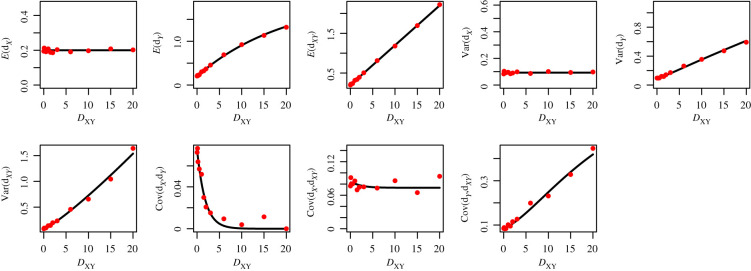
Assessing the accuracy of average pairwise difference results, 2*μη*_*X*_ = 1, *η*_*Y*_ = 10*η*_*X*_. We compare our theoretical results based on coalescence theory using equations presented here (black line) with empirical estimates using 1500 independently simulated gene sequences (red dots), *n*_*X*_ = *n*_*Y*_ = 10 sampled individuals. (Online version in colour.)

We compare three population size change models, denoted by *η*_*Y*_ = 1*η*_*X*_, *η*_*Y*_ = 2*η*_*X*_ and *η*_*Y*_ = 10*η*_*X*_, along with three mutation rates 2*μη*_*X*_ = 10, 1, 0.1, for a total of nine simulation scenarios. We present one of those scenarios here (figure 6), and leave the full set of results to the electronic supplementary material, figures S2–S10. While allowing for variance in the empirical estimates from sample size, coalescent and mutational variation, there is strong agreement between the theoretical and simulated results. Note that the theoretical quantities assume an infinite-sites model of mutation, whereas our simulations are performed assuming a realistic, finite-sites model (1500 independent genes of 10 000 bp each; see electronic supplementary material for full simulation details). We choose to compare this finite-sites model over simulations using a model of infinite sites to demonstrate the applicability of the results to the types of data that will be used in practice, and to demonstrate when there are limitations. We leave a demonstration of the accuracy of our variance/covariance calculations in relation to the previous results derived for constant population size in Takahata & Nei [7] to the electronic supplementary material, §C.

## 7. Accuracy in estimating *F*_ST_

A direct extension of our discussion on the mean and variance of average pairwise nucleotide differences is to the measurement *F*_ST_ for a given species tree, mutation rate and sample size. Slatkin (1991, equation 8) [11] presented a coalescent-based definition of *F*_ST_ as a function of the difference in expected time to coalescence for a collection of subpopulations. Specializing to two sub populations of interest, *X* and *Y*, Slatkin’s *F*_ST_ can be expressed as

7.1 $$F_{\mathrm{ST}}=\frac{E(t_{i,j})-(1/2)\left(E(t_{i,i^{\prime }})+E(t_{\;j,j^{\prime }})\right)}{E(t_{i,j})},$$

where *i*, *i*′ are from population *X*, and *j*, *j*′ are individuals sampled from population *Y*. This definition of *F*_ST_ relies on a ratio of estimates of average coalescence times, where average pairwise differences in DNA sequence data are used as the proxy to estimate the unknown coalescence times. Discussed in Slatkin and Hudson *et al.* [11,26], for two populations *X* and *Y*, *F*_ST_ can be estimated from a non-recombining portion of the genome using

7.2 $$F_{\mathrm{ST}}\approx \frac{d_{XY}-(1/2)(d_{X}+d_{Y})}{d_{XY}}\overset{\mathrm{define}}{=}F_{\mathrm{ST}}^{G}.$$

For the sake of this paper, we differentiate *F*_ST_ and $F_{\mathrm{ST}}^{G}$ as the exact measurement from unobservable coalescence times and the estimate from pairwise differences across multiple sequences, respectively. As we have shown above, the expectation, variance and covariance of these sample average pairwise differences contained in equation (7.2) can be derived using coalescent theory, for a given mutation parameter *μ* and sample sizes. We can use these to study the accuracy of the $F_{\mathrm{ST}}^{G}$ estimator to Slatkin’s *F*_ST_ under an arbitrary species tree, S.

To begin, it is important to note that the mean of a ratio is not the ratio of means, specifically it is the case that

7.3 $$\begin{array}{rl} E(F_{\mathrm{ST}}^{G}) & \neq \frac{E(d_{XY})-(1/2)\left(E(d_{X})+E(d_{Y})\right)}{E(d_{XY})}\\ & =\frac{2\mu E(t_{i,j})+\mu \left(E(t_{i,i^{\prime }})+E(t_{\;j,j^{\prime }})\right)}{2\mu E(t_{i,j})}=F_{\mathrm{ST}}. \end{array}$$

This implies that the estimator $F_{\mathrm{ST}}^{G}$ is potentially a biased estimator of *F*_ST_, such that $F_{\mathrm{ST}}-E(F_{\mathrm{ST}}^{G})\neq 0$. To study this bias, we need an expression for the mean of $F_{\mathrm{ST}}^{G}$. In general, there is no closed form for the mean of a ratio of dependent random variables, so we will first simplify our terms, and then approximate the mean and variance using a Taylor expansion. We can first simplify the expressions for $E(F_{\mathrm{ST}}^{G})$

7.4 $$E(F_{\mathrm{ST}}^{G})=E\left(\frac{d_{XY}-(1/2)(d_{X}+d_{Y})}{d_{XY}}\right)=1-\frac{1}{2}E\left(\frac{d_{X}+d_{Y}}{d_{XY}}\right)$$

and

7.5 $$\text{Var}(F_{\mathrm{ST}}^{G})=\text{Var}\left(\frac{d_{XY}-\frac{1}{2}(d_{X}+d_{Y})}{d_{XY}}\right)=\frac{1}{4}\text{Var}\left(\frac{d_{X}+d_{Y}}{d_{XY}}\right).$$

We are now interested in the mean and variance of the ratio (*d*_*X*_ + *d*_*Y*_)/*d*_*XY*_. As generally discussed in Stuart & Kendall [28], we can use a second-order Taylor expansion of *f*(*A*, *B*) = *A*/*B* around the mean values $\left(E(d_{X})+E(d_{Y}),E(d_{XY})\right)$ to get an approximation to the mean, and a first-order expansion around the means to get an approximation of the variance of the ratio term. We can approximate the mean as

7.6 $$\begin{array}{rl} E\left(\frac{d_{X}+d_{Y}}{d_{XY}}\right) & \approx \frac{E(d_{X})+E(d_{Y})}{E(d_{XY})}+\frac{E(d_{X})+E(d_{Y})}{E{\left(d_{XY}\right)}^{3}}\mathrm{Var}(d_{XY})\\ & \;-\frac{1}{E{\left(d_{XY}\right)}^{2}}\left[\mathrm{Cov}(d_{X},d_{XY})+\mathrm{Cov}(d_{Y},d_{XY})\right]. \end{array}$$

By rearranging terms, observe that $E(F_{\mathrm{ST}}^{G})$ is a function of *F*_ST_, along with other mean, variance and covariance terms

7.7 $$\begin{array}{rl} E(F_{\mathrm{ST}}^{G}) & =1-\frac{1}{2}E\left(\frac{d_{X}+d_{Y}}{d_{XY}}\right)\\ & \approx F_{\mathrm{ST}}+\frac{1}{2E{\left(d_{XY}\right)}^{2}}(\mathrm{Cov}(d_{X},d_{XY})+\mathrm{Cov}(d_{Y},d_{XY})\\ & \;-\frac{E(d_{X})+E(d_{Y})}{E(d_{XY})}\mathrm{Var}(d_{XY})). \end{array}$$

Using this, we can get an expression for the bias of $E(F_{\mathrm{ST}}^{G})$

7.8 $$\begin{array}{rl} E(F_{\mathrm{ST}}^{G})-F_{\mathrm{ST}} & \approx \frac{1}{2E{\left(d_{XY}\right)}^{2}}(\mathrm{Cov}(d_{X},d_{XY})+\mathrm{Cov}(d_{Y},d_{XY})\\ & \;-\frac{E(d_{X})+E(d_{Y})}{E(d_{XY})}\mathrm{Var}(d_{XY})). \end{array}$$

Similarly, we can get a first-order approximation for the variance of $F_{\mathrm{ST}}^{G}$:

7.9 $$\begin{array}{rl} \text{Var}(F_{\mathrm{ST}}^{G}) & =\frac{1}{4}\text{Var}\left(\frac{d_{X}+d_{Y}}{d_{XY}}\right)\\ & \approx \frac{1}{4}(\frac{\mathrm{Var}(d_{X})+\mathrm{Var}(d_{Y})+2\mathrm{Cov}(d_{X},d_{Y})}{{\left(E(d_{X})+E(d_{Y})\right)}^{2}}\\ & +\frac{{\left(E(d_{X})+E(d_{Y})\right)}^{2}}{E{\left(d_{XY}\right)}^{4}}\mathrm{Var}(d_{XY})\\ & -2\frac{E(d_{X})+E(d_{Y})}{E{\left(d_{XY}\right)}^{3}}\left(\mathrm{Cov}(d_{X},d_{XY})+\mathrm{Cov}(d_{Y},d_{XY})\right)). \end{array}$$

**Figure 7.**
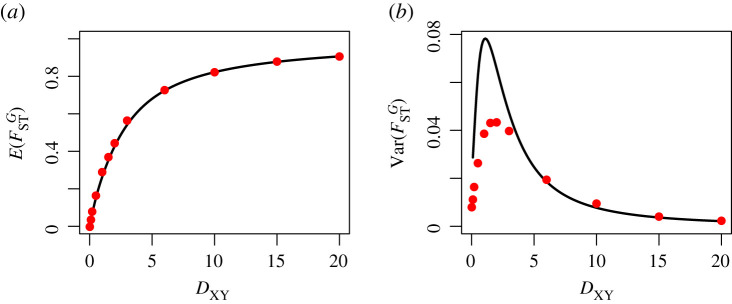
Accuracy of approximations to the mean and variance of $F_{\mathrm{ST}}^{G}$, 2*μη*_*X*_ = 1, *η*_*Y*_ = *η*_*X*_. A comparison of the approximations in equations (7.7) and (7.9) (black curves) to values estimated from empirical simulations (red dots). (*a*) The approximated value to $E(F_{\mathrm{ST}}^{G})$ as a function of divergence time, *D*_*XY*_, for equal sample sizes *n*_*X*_, *n*_*Y*_ accurately approximates simulated estimates. (*b*) The first-order approximation for the variance $\mathrm{Var}(F_{\mathrm{ST}}^{G})$ as a function of *D*_*XY*_ is a poor approximation for more recent divergence time models. (Online version in colour.)

Figure 7 shows the accuracy of the two Taylor approximations under a constant population size model for mutation rate 2*μη*_*X*_ = 1. The approximation for the mean is a good one, however the first-order approximation to the variance is insufficient for low divergence times, as it can be seen there are higher-order terms involved. From this, we decide that we cannot approximate the variance in $F_{\mathrm{ST}}^{G}$ well with this method, and do not pursue this aspect further. Electronic supplementary material, figures S11 and S12, demonstrate the accuracy of the Taylor approximations under alternate mutation rates, and it can be seen that the approximation to $E(F_{\mathrm{ST}}^{G})$ breaks down under a 10× reduction in the mutation rate (2*μη*_*X*_ = 0.1) due to the high variance in estimating variance/covariance terms of the *d* statistics.

In what follows, we will evaluate the bias in the $F_{\mathrm{ST}}^{G}$ estimator of *F*_ST_ under different demographic and genetic parameters, using the approximation given in equation (7.7).

### (a) Results for the mean and bias of $F_{\mathrm{ST}}^{G}$

In this section, we study the effects of varying demographic and genetic parameters on the expectation of $F_{\mathrm{ST}}^{G}$ and consequently its bias as an estimator of *F*_ST_. First, we start with a discussion on the differences between $E(F_{\mathrm{ST}}^{G})$ and *F*_ST_, both as described above. Supposing we knew the true values, we calculate *F*_ST_ using only the individual expectations of *d*_*X*_, *d*_*Y*_ and *d*_*XY*_. We can write

7.10 $$\begin{array}{rl} F_{\mathrm{ST}} & =\frac{E(d_{XY})-(1/2)\left(E(d_{X})+E(d_{Y})\right)}{E(d_{XY})}=1-\frac{1}{2}\frac{E(d_{X})+E(d_{Y})}{E(d_{XY})}\\ & =1-\frac{1}{2}\frac{E(t_{i,i^{\prime }})+E(t_{\;j,j^{\prime }})}{E(t_{i,j})}. \end{array}$$

Immediately we can note that *F*_ST_ is not dependent on sample sizes *n*_*X*_, *n*_*Y*_ or the mutation rate, *μ*. Instead, it is solely a function of mean coalescence times, and is only variable in the demographic parameter space. Also, note the fundamental difference between $E(F_{\mathrm{ST}}^{G})$ and *F*_ST_ is the term

7.11 $$E\left(\frac{d_{X}+d_{Y}}{d_{XY}}\right)\text{ versus }\frac{E(d_{X})+E(d_{Y})}{E(d_{XY})}.$$

It is known that ratio estimators are in general biased [29]. Jensen’s inequality [30] tells us, for a convex function *f*(*t*), that

7.12 E( f (t))≥f(E(t)).

Letting *f*(*t*) = (*d*_*X*_ + *d*_*Y*_)/*d*_*XY*_ and observing that *d*_*XY*_ ≥ 1/2(*d*_*X*_ + *d*_*Y*_), the inequality implies

7.13 $$E\left(\frac{d_{X}+d_{Y}}{d_{XY}}\right)\geq \frac{E(d_{X})+E(d_{Y})}{E(d_{XY})}.$$

**Figure 8.**
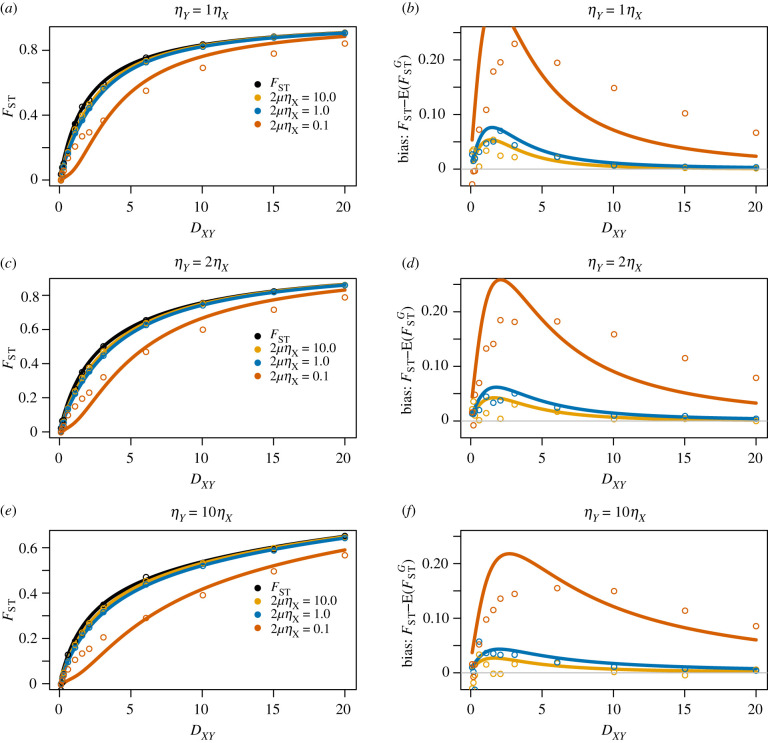
*F*_ST_ approximation bias using $E(F_{\mathrm{ST}}^{G})$ across divergence times. Under varying population size scenarios (rows), we study the difference between theoretical *F*_ST_ and the expected estimate calculated from pairwise differences, $E(F_{\mathrm{ST}}^{G})$, to highlight the potential biases in doing so. (*a*,*c*,*e*) On the *y*-axis are values $E(F_{\mathrm{ST}}^{G})$ and *F*_ST_ as functions of divergence time *D*_*XY*_. We plot the true value of *F*_ST_ in black, and approximations $E(F_{\mathrm{ST}}^{G})$ using equation (7.7) under three mutation rates. (*b*,*d*,*e*) The difference between the true *F*_ST_ (black line in adjacent plot) and the expected sample quantity, to represent the bias in estimation. We simulated assuming equal sample sizes *n*_*X*_ = *n*_*Y*_ = 10. In all figures, dots represent simulated estimates from 1500 independent genes. (Online version in colour.)

Thus we expect $E(F_{\mathrm{ST}}^{G})$ to be a negatively biased estimate of *F*_ST_. As the divergence time between *X* and *Y* becomes deeper (more ancient), we expect *d*_*X*_ + *d*_*Y*_ to become increasingly independent from *d*_*XY*_ and $E(F_{\mathrm{ST}}^{G})$ to become increasingly closer to *F*_ST_. Also, letting the number of mutations increase in an infinite-sites model, the estimates of *d*_*X*_, *d*_*Y*_ and *d*_*XY*_ become closer to their expectations, bringing equation (7.13) closer to equality. Figure 8 demonstrates the relationship between $E(F_{\mathrm{ST}}^{G})$ and *F*_ST_ under varying divergence times *D*_*XY*_, population sizes and mutation rates *μ*. As discussed above, the relative bias of $F_{\mathrm{ST}}^{G}$ is much less under a deep divergence model (*D*_*XY*_ = 20.0, in units of 2*η*_*X*_ generations) as *d*_*X*_, *d*_*Y*_ and *d*_*XY*_ are more independent, compared to a more shallow divergence (*D*_*XY*_ = 1.0), where we see in our example *F*_ST_ is three times as large as $E(F_{\mathrm{ST}}^{G}|2\mu \eta_{X}=0.1)$. It is clear that $F_{\mathrm{ST}}^{G}$ is a faithful estimator of *F*_ST_ under very high mutation rates, however, it is biased downward for small values of *μ*, although the bias is reduced for deep divergence models. When estimating *F*_ST_ from multiple genes across the genome, one approach used to reduce the estimation bias is to estimate each term in equation (7.10) individually and apply a ‘ratio of averages’ approach [31], as further highlighted in the discussion.

### (b) Effect of bottleneck timing on *F*_ST_

**Figure 9.**
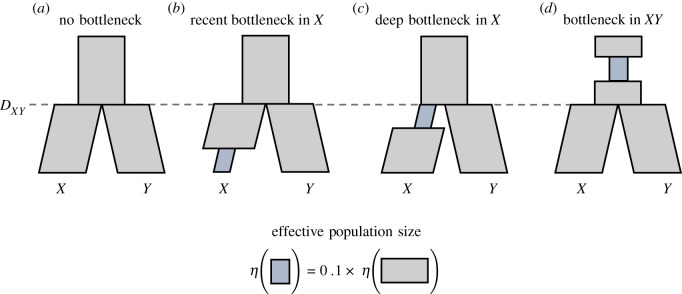
Bottlenecks considered in a tree of two species. (*a*) Constant population size tree with no bottleneck. (*b*) A bottleneck occurring in the recent history of species *X*. (*c*) A bottleneck which occurred directly after speciation in population *X*. (*d*) A bottleneck which occurred in the population ancestral to both *X* and *Y*. In all trees, the non-bottleneck population sizes are a fixed constant. (Online version in colour.)

**Figure 10.**
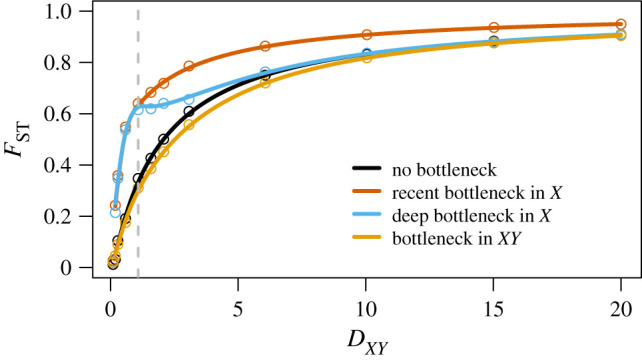
The effect of different population bottlenecks on *F*_ST_. Four different bottleneck scenarios were considered in the genetic history of two species *X* and *Y*, as described in figure 9. Curves represent theoretical results from STCov, open circles are empirical estimates from 1500 independently simulated sequences under 2*μη*_*X*_ = 1.0 mutation rate. Note that as bottleneck lengths in *X* were fixed to be min (1.0, *D*_*XY*_), for *D*_*XY*_ ≤ 1, the population histories of recent and deep bottlenecks in *X* are identical. The vertical dashed line at *D*_*XY*_ = 1 indicates this boundary. (Online version in colour.)

Population bottlenecks can drastically affect the genetic diversity of populations over evolutionarily short periods of time. In the context of *F*_ST_, the question of when a bottleneck occurred in a history of evolution is key in understanding its impact on population differentiation. In this section, we use the flexibility of STCov to explore the effect of a population bottleneck placed at various times in the history of two theoretical species, *X* and *Y*, on *F*_ST_. Here, we model a population bottleneck as a 10 × reduction in the population size *η*_0_ for a fixed length of time (1.0 in units of 2*η*_0_ generations). We study four scenarios as described in figure 9. For varying divergence times *D*_*XY*_, we use STCov to calculate *F*_ST_ under each scenario, and use empirical simulations via ms and SeqGen to validate our results. We find that a recent bottleneck has the largest impact on *F*_ST_ at every divergence time tested (figure 10), demonstrating an increased level of differentiation as compared to the scenario with no bottleneck. Both scenarios of deeper bottlenecks have much less effect on overall *F*_ST_ despite their bottlenecks being identical in size and length. This illustrates that the timing of variation-reducing events such as a bottleneck plays a large role in the impact to measured genetic differentiation using *F*_ST_, where the impact can be effectively lost given sufficient time post-bottleneck.

### (c) Bias in the *F*_ST_ estimator for gene flow

The value of *F*_ST_ is often used to estimate levels of gene flow between populations. Wright [32] first derived the relationship between *F*_ST_ to estimate *Nm* in an Island model, where *N* is the number of individuals in each deme (sub-population), and *m* is the fraction of migrants into the deme in each generation. Hudson *et al.* [26] used this relationship to estimate *Nm* using the following expression:

7.14 $${\left\langle Nm\right\rangle }_{F}=\frac{1}{2}\left(\frac{1}{F_{\mathrm{ST}}}-1\right),$$

where *F*_ST_ is an estimate from sequence data, i.e. $F_{\mathrm{ST}}^{G}$ in our notation. The results of the simulations presented there show estimates using 〈*Nm*〉_*F*_ are upward-biased using an estimate of *F*_ST_ from sequence data in place of the unknown *F*_ST_ based on coalescence times. There are two potential sources of this bias, the estimator function, 〈*Nm*〉_*F*_, and the estimate, $F_{\mathrm{ST}}^{G}$. The scope of this study concerns the role of estimator $F_{\mathrm{ST}}^{G}$, and we can investigate the effect of this estimator compared to using the true value, *F*_ST_. We note that we do not intend to estimate or study gene flow in this manuscript, but simply evaluate the accuracy of the function 〈*Nm*〉_*F*_ when an estimate of *F*_ST_ is used.

To start, we can once again use a Taylor expansion to get an approximation for the expected value of 〈*Nm*〉_*F*_, when using $F_{\mathrm{ST}}^{G}$

7.15 $$\begin{array}{rl} E({\left\langle Nm\right\rangle }_{F}) & =\frac{1}{4}E\left(\frac{d_{X}+d_{Y}}{d_{XY}-(1/2)(d_{X}+d_{Y})}\right)\approx \frac{1}{4}\frac{E(d_{X})+E(d_{Y})}{E(d_{XY})-(1/2)\left(E(d_{X})+E(d_{Y})\right)}\\ & \;\times [1-\frac{\mathrm{Cov}(d_{X},d_{XY})+\mathrm{Cov}(d_{Y},d_{XY})-(1/2)\left(\mathrm{Var}(d_{X})+\mathrm{Var}(d_{Y})\right)-\mathrm{Cov}(d_{X},d_{Y})}{\left(E(d_{X})+E(d_{Y})\right)\left(E(d_{XY})-(1/2)\left(E(d_{X})-E(d_{Y})\right)\right)}\\ & \;+\frac{\mathrm{Var}(d_{XY})+(1/4)\left(\mathrm{Var}(d_{X})+\mathrm{Var}(d_{Y})+2\mathrm{Cov}(d_{X},d_{Y})\right)-\mathrm{Cov}(d_{X},d_{XY})-\mathrm{Cov}(d_{Y},d_{XY})}{{\left(E(d_{XY})-(1/2)\left(E(d_{X})-E(d_{Y})\right)\right)}^{2}}]. \end{array}$$

**Figure 11.**
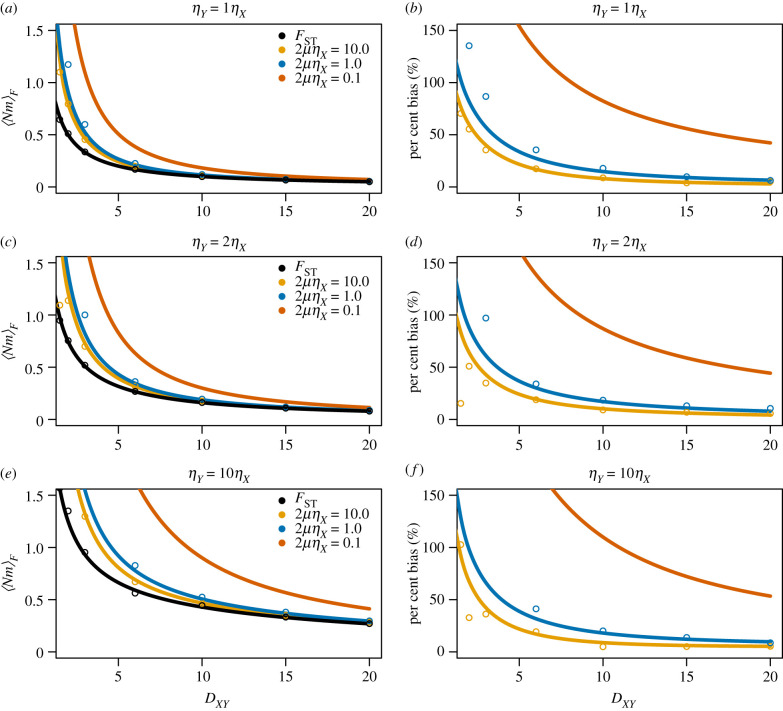
〈*Nm*〉_*F*_ approximation bias across divergence times and mutation rates. Under varying population size scenarios (rows), we demonstrate the difference between theoretical 〈*Nm*〉_*F*_ and the expected estimate when calculating from pairwise differences using equation 7.15. (*a*,*c*,*e*) On the *y*-axis are values 〈*Nm*〉_*F*_ as functions of divergence time *D*_*XY*_. We plot the value when using the true *F*_ST_, and approximations $E({\left\langle Nm\right\rangle }_{F}|2\mu \eta_{X})$, for mutation rates 2*μη*_*X*_ = 10.0,1.0 and 0.1. (*b*,*d*,*f*) The per cent difference between 〈*Nm*〉_*F*_ using *F*_ST_ (black line in *a*,*c*,*e*) and the expected sample quantity to represent the bias in estimation. We simulated assuming equal sample sizes *n*_*X*_ = *n*_*Y*_ = 10, and population size structure as indicated at the top of each plot. For a fixed sample size, the expected sample quantity tends to overestimate the ‘true’ value, with the amount of overestimation a function of *μ* and *D*_*XY*_.

We can use this expression to study the difference between using the estimator, $F_{\mathrm{ST}}^{G}$, and the (unknown) true value, *F*_ST_, in the expression for 〈*Nm*〉_*F*_. Figure 11 shows the difference between using *F*_ST_ and $F_{\mathrm{ST}}^{G}$ in 〈*Nm*〉_*F*_ under different mutation rates, population sizes and species divergence times. From the figure, we see that the expectations are, in fact, overestimates. In this figure, 10 individuals are sampled from each population. When the divergence time *D*_*XY*_ is low, the bias relative to the true value is substantial, resulting in an estimate twice as large as that which would have been obtained using an accurate estimate of *F*_ST_. For high mutation rates, this bias decreases rapidly as *D*_*XY*_ increases. For a low mutation rate, 2*μη*_*X*_ = 0.01, a bias of greater than 50% overestimation persists. Even at high mutation rates, an upwards bias of about approximately 5% exists even at large divergence time values. Note, however, that we do not see a large difference in the bias across different population size models. The results here can explain (at least a portion of) the bias seen in Hudson *et al.* [26], that using an estimate of *F*_ST_ can result in an artificial increase in the function 〈*Nm*〉_*F*_.

### (d) Accuracy of log transform for linearizing *F*_ST_

Under a neutral divergence model, *F*_ST_ has also commonly been transformed as a linear approximation to the population divergence time, *D*_*XY*_. Discussed in Cavalli–Sforza [25], and later Nielsen *et al.* [24], is that given an estimate of *F*_ST_, *D*_*XY*_ can be estimated by the transformation

7.16 $${\hat{D}}_{XY}\propto -log(1-F_{\mathrm{ST}}^{G}).$$

Another commonly used transformation, presented in Slatkin [33], relates the time of divergence to a ratio of *F*_ST_ values

7.17 $${\hat{D}}_{XY}\propto \frac{F_{\mathrm{ST}}^{G}}{1-F_{\mathrm{ST}}^{G}}.$$

Here, we evaluate the accuracy of these transformations by approximating the expected value of each using similar Taylor expansions, as earlier. Without having an accurate approximation of $\mathrm{Var}(F_{\mathrm{ST}}^{G})$, we can only make a first-order approximation of equation (7.16) such that

7.18 $$E(-\log(1-F_{\mathrm{ST}}^{G}))\approx -\log(1-E(F_{\mathrm{ST}}^{G})).$$

For equation (7.17), by plugging in the estimator for *F*_ST_ from equation (7.2), we find

$$\frac{F_{\mathrm{ST}}^{G}}{1-F_{\mathrm{ST}}^{G}}=2\frac{d_{XY}}{d_{X}+d_{Y}}-1.$$

Taking the expectation of this quantity

7.19 $$E\left(\frac{F_{\mathrm{ST}}^{G}}{1-F_{\mathrm{ST}}^{G}}\right)=2E\left(\frac{d_{XY}}{d_{X}+d_{Y}}\right)-1.$$

By deriving a similar second-order Taylor approximation for the expectation on the right-hand side, as we did earlier with $E(\left(d_{X}+d_{Y}\right)/d_{XY})$, we get

7.20 $$\begin{array}{rl} E\left(\frac{d_{XY}}{d_{X}+d_{Y}}\right) & \approx \frac{E(d_{XY})}{E(d_{X})+E(d_{Y})}-\frac{\mathrm{Cov}(d_{X},d_{XY})+\mathrm{Cov}(d_{Y},d_{XY})}{{\left(E(d_{X})+E(d_{Y})\right)}^{2}}\\ & \;+\frac{\mathrm{Var}(d_{X})+\mathrm{Var}(d_{Y})+2\mathrm{Cov}(d_{X},d_{Y})}{{\left(E(d_{X})+E(d_{Y})\right)}^{3}}E(d_{XY}), \end{array}$$

and we have a second-order Taylor approximation of the expectation of equation (7.17).

**Figure 12.**
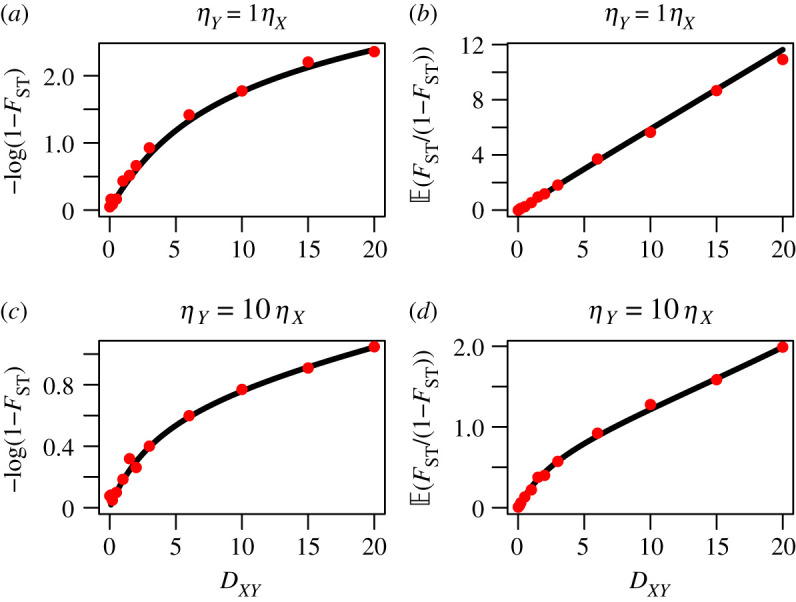
Linearized *F*_ST_ estimates. Testing the linearity of two *F*_ST_ transformations plotted against species divergence time, *D*_*XY*_. On the left (*a*,*c*) is the approximate mean log transformed value. On the right (*b*,*d*) is the approximated mean fraction transformed value. Both use $F_{\mathrm{ST}}^{G}$ as a proxy for the unknown *F*_ST_. Plotted on the *x*-axis of all is the simulated divergence time. The red circles correspond to empirical values of $E(-\log(1-F_{\mathrm{ST}}))$ and $E(F_{\mathrm{ST}}/(1-F_{\mathrm{ST}}))$ to verify the accuracy of the approximation (line in black). (*a*,*b*) correspond to the approximations under a constant population size model. (*c*,*d*) correspond to the *η*_*Y*_ = 10*η*_*X*_ imbalanced population size model. (Online version in colour.)

In figure 12, we evaluate the linearity between these expressions and divergence time, and the accuracy of our approximations against simulated data (line versus dots), under two different population size models. It is clear that Slatkin’s [33] linear *F*_ST_ is a linear predictor of divergence time under the constant population size model assumed in its derivation. However, under a model where the population size of species *Y* is 10 times higher than *X*, the linearity expectedly disappears. The log transformation of Nielsen *et al.* and Cavalli-Sforza [24,25] performs worse and can only be used as a local-linear approximation. Across large values of *D*_*XY*_, it demonstrates clear nonlinear behaviour and Slatkin’s [33] transformation is preferable under the conditions investigated here.

## 8. Discussion

In this study, we have derived the equations and recursions needed to calculate exact values for the covariance between pairs of coalescence times in a species tree model, allowing for piecewise constant changes in population sizes throughout the tree. Using these expressions, we are able to build on previous theory to get exact values for the mean, variance and covariance of the average number of pairwise differences for a given mutation rate and sample size. We have demonstrated that in the constant population size scenario, we can exactly recreate the covariance results of Takahata & Nei [7]. The equations and recursions derived here are implemented in a freely available software package, STCov, which allows for exact calculations under any piecewise constant model of divergence for arbitrary numbers of species/populations. While the covariance results presented here are interesting on their own, we imagine there are many further applications of the summary statistics presented here.

One such application we explored is the properties of Slatkin’s *F*_ST_ and its approximation using sequence data, $F_{\mathrm{ST}}^{G}$, under a divergence model. Under the infinite-sites model with no recombination, we demonstrate the known negative bias in estimating *F*_ST_ using sequence data and the ‘average of ratios’ approach. We show that the magnitude of the bias is a function of both mutation rate and population divergence time, with the amount of bias decreasing as both mutation rates and divergence times increase. The bias, however, is non-vanishing for low mutation rates, even as simulated divergence time increases, and is further exaggerated for imbalanced population sizes. As such, the results of the transformation for *F*_ST_ used for gene-flow estimation can be biased upwards when using empirical estimates, which reaffirms discussion in Hudson *et al.* [26] and provides further insight to the source of the bias. We therefore advocate that when looking at *F*_ST_ in a gene-by-gene fashion, such as when performing local *F*_ST_ scans, to consider that empirical estimates of Slatkin’s *F*_ST_ are generally accurate for high values of mutation and deep divergence, but warn against its over-interpretation in low mutation or recent divergence scenarios, where the *F*_ST_ estimate can be uninformative. We recommend using equation (7.8) to estimate the expected level of bias upon application.

Throughout the theoretical equations presented here, we assumed an infinite-sites model of mutation with no recombination between sites. However, allowing for recombination between sites provides more stable estimates of the expectations of pairwise differences. As discussed in Wakeley [15], allowing for an increasing amount of recombination between loci decreases the error in estimates of expectations of *d*_*X*_, *d*_*Y*_ and *d*_*XY*_. At the limit of infinitely free recombination between loci, estimates of equation (7.13) tend towards equality and thus the estimator $E(F_{\mathrm{ST}}^{G})$ would converge to the value of *F*_ST_, mitigating the negative bias seen here. Therefore, aligning with conclusions drawn in Bhatia *et al.* [31], in the age of whole-genome estimates of *F*_ST_, taking a ‘ratio of averages’ across independent loci rather than the ‘average of ratios’ approach to *F*_ST_ can sidestep the bias we have presented when estimating *F*_ST_ from loci across an entire genome; the former also having the advantage of being a more numerically stable estimator.

Independent of bias, our equations demonstrate that the timing of a bottleneck can drastically impact measured levels of *F*_ST_. Specifically, that the impact of population variation can vanish given enough time. Finally, we study the accuracy of a couple of commonly used linear transformations of *F*_ST_ as approximate measures of population divergence times, and find, for equal population sizes, the estimator proposed in Slatkin [33] has the best performance, but when population sizes are no longer equal, expectedly, even this transformation shows deviations from linearity.

There are many interesting properties to study with the covariance of pairwise coalescent times and pairwise differences. We hope that the software provided, STCov, will allow for further investigation into the properties and usefulness of these quantities for estimating various aspects of species trees, such as topology reconstruction, divergence time and population size estimation, gene flow and admixture detection.

## 9. Software availability

Along with this manuscript, we provide software (implemented in C++) freely available for download which calculates the various coalescent quantities presented here (means, variances, covariances and shared branch length). We have designed the code to be very flexible to user inputted species trees. The program outputs exact quantities for any user-defined rooted, bifurcating, piecewise-constant population size species tree. Download the code at https://github.com/gaguerra/STCov.

## Data Availability

All scripts used in this study are openly accessible through https://github.com/StochasticBiology/boolean-efflux.git. The data are provided in electronic supplementary material [34].
